# Computational study of blood flow inside MCA aneurysm with/without endovascular coiling

**DOI:** 10.1038/s41598-023-31522-x

**Published:** 2023-03-20

**Authors:** Asal Sadeh, Admin Kazemi, Moharam Bahramkhoo, M. Barzegar Gerdroodbary

**Affiliations:** 1 Department of Mechanical Engineering, Islamic Azad University, Bandar Anzali, Iran; 2 Department of Mechanical Engineering, Babol Noshirvani University of Technology, Babol, Iran

**Keywords:** Biomedical engineering, Mechanical engineering

## Abstract

The simulation of blood hemodynamics inside the MCA aneurysm is done to investigate the potential region for rupture and hemorrhage. The main focus of this work is to disclose the impacts of endovascular coiling on blood hemodynamics and the risk of aneurysm rupture. Navier–stokes equations are solved for the computational study of blood flow while it is assumed that flow remains laminar, unsteady, and non-Newtonian. Influences of blood hematocrits and coiling porosity are also examined in this work. Obtained results show that impacts of blood hematocrit on the maximum OSI are limited in the MCA case.

## Introduction

It was found that the creation of these aneurysms is mainly related to exclusive structural aspects of the cerebral vasculature^1,2^. In fact, the media of classical saccular aneurysm is very thin and in most cases, the internal elastic lamina is not presented^3–5^. Therefore, the wall of artery consists of the adventitia and intima. Hence, the aneurysm wall is mainly composed of layered collagen while it should withstand sever pressure of the blood inside the sac. The multidirectional collagen fibres are the key aspects of an aneurysm wall and the collagen fibres turn into straight under physiologic pressures, and thus determine the total stiffness of the lesion^6–8^. Fluid shear stress moderates endothelial cell reshaping via elongation and realignment, and the rates of remodelling endothelial cells is associated with the time variation of wall shear stress (WSS)^5,9,10^.

So, hemodynamic factors, for instance WSS, blood pressure, velocity, and particle residence time, play significant roles in the growing and breach of cerebral aneurysms^11–13^. Besides, the shape and size of aneurysm as well as the aneurysm's connection to the parent vessel, aspect ratio, and volume (depth/neck width) significantly influences on aneurysm hemodynamic^14–16^.

Among these characteristics, WSS is one of the main factors that determine the growth, development and rupture of the cerebral aneurysms. Several previous works demonstrated that the main parameter for the growth of cerebral aneurysm is high WSS^17,18^. It is also reported that the rupture of the aneurysm occurs due to low level of WSS which is related to low blood flow situation^19,20^. the accumulation of red blood cells, in addition to the adhesion and aggregation of both leukocytes and platelets along the intimal surface occurs when blood flow locally stagnated. Indeed, high WSS results in mechanical stimulation and consequently, dysfunction of flow-induced nitric oxide occurs in this condition^21–23^. According to previous studies, to maintain the feature of the aneurysmal wall, WSS of 2 Pa is appropriate in which deterioration of endothelial cells is noticed in the lower WSS. Hence, fluid dynamic studies of blood stream are significant to find quantitative measures for the treatment of saccular aneurysms^24–26^. In prior research, three-dimensional (3D) computerized angiography is done to investigate flow feature in acrylic models of cerebral aneurysm and reported non-homogenous WSS distribution over aneurysm wall.

**Figure 1:**
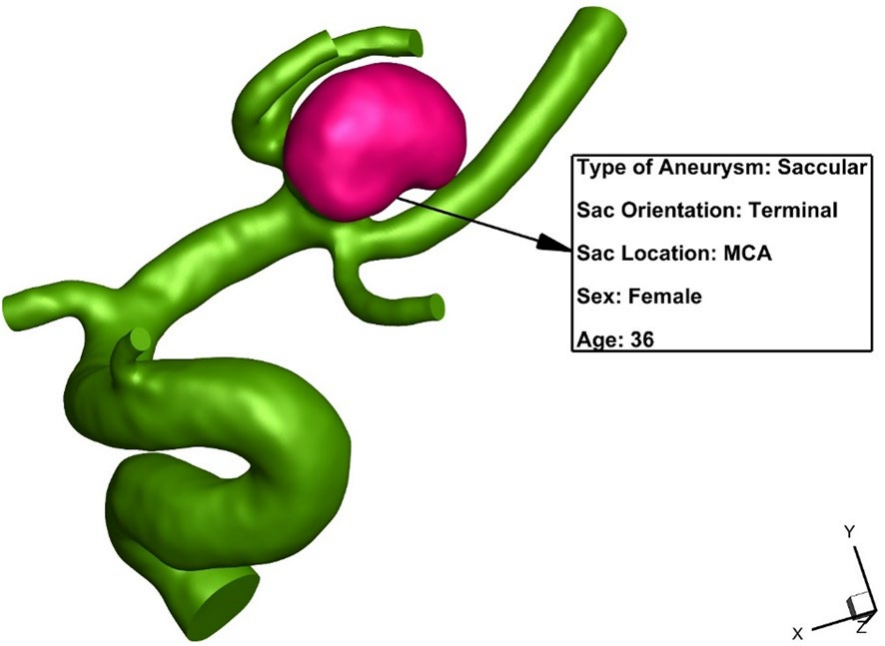
MCA Aneurysm.

In this article, a numerical simulation of the blood streaming inside the MCA aneurysm is done to study the effects of hemodynamic parameters on the risk of aneurysm rupture. The real 3-D shape of an MCA aneurysm (Fig. 1) is selected for this investigation. The effects of endovascular coiling are also investigated on the hemodynamic characteristics. Pressure distribution and blood velocity contour for the chosen sac are also compared in different coiling porosities.

## Governing equations and applied methods

It is confirming that all methods were carried out in accordance with relevant guidelines and regulations. Besides, all experimental protocols were approved by a named institutional and/or licensing committee and it is confirmed that informed consent was obtained from all subjects and/or their legal guardian(s).

**Figure 2:**
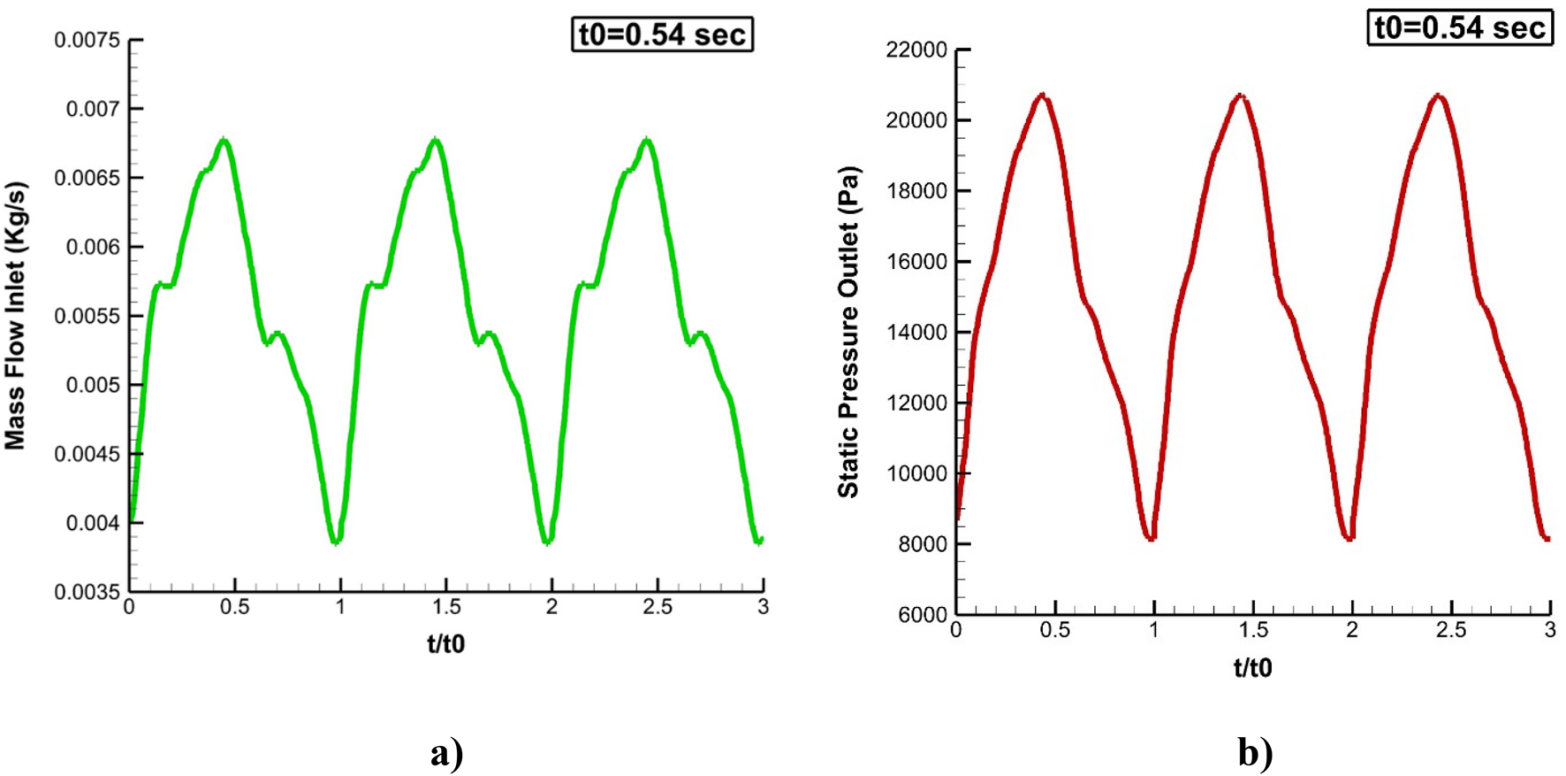
Applied boundary condition.

Computational fluid dynamic is known as reliable method for modeling of flow in engineering applications^27–30^. The simulation of the bloodstream is done by solving Navier–Stocks (NS) equations while it is assumed that blood flow is Non-Newtonian, laminar, and transient^22–24^. Simple algorithm is used for solving of governing equations^31–34^. This algorithm is efficient method for the flow stream with lo velocity and viscous flow^35–39^. Due to the pulsatile flow of blood, simulations are done for three cardiac cycles, and the inlet and outlet velocity profile is applied as presented in Fig. 2^25^. For estimation of the blood viscosity, the Casson model is used in the present work^26^.

As mentioned, the influence of the coiling endovascular technique is investigated in this research. To do this, the sac domain is assumed filled with porous medium. To equivalent the porosity of coiling, the permeability (k) factor is calculated since this indicates the surface area to volume ratio^26^. Table 1 presents the details of applied permeability for the two selected porosities. Figure 1 also presented geometric details of selected MCA aneurysms. Volume of aneurysm, Length and diameter of Coil are 103.87 (mm^3^), 30 (cm) and 0.254 (mm), respectively.

**Table 1:** Porosity condition.

Porosity	Permeability (m^2^)	1/Permeability (1/m^2^)
0.75	3.7E−08	2.70E+07
0.85	5.4E−08	1.85E+07

**Figure 3:**
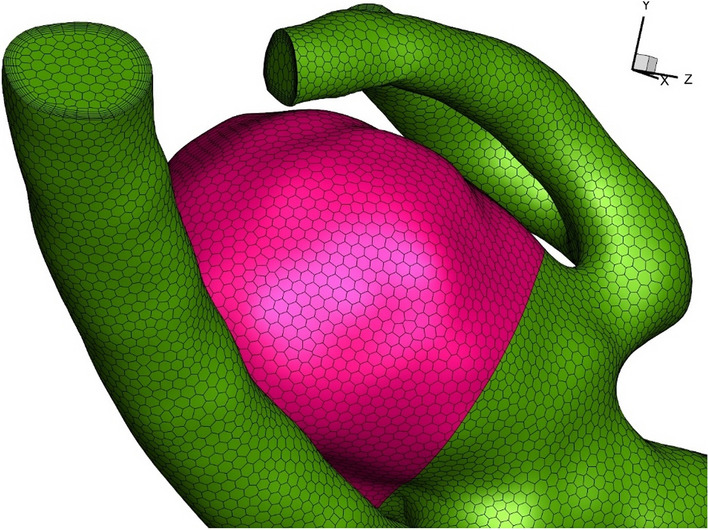
Generated grid.

Mesh production for the selected model is a significant step for the computational modeling^40–45^. Figure 3 demonstrates the produced grid. As shown in this figure, the boundary layer is applied on the sac and vessel wall to increase the precision of the results. Uniform grids are produced inside the domain and the distribution of the grid inside the sac and parent vessel is almost homogeny. Grid study is also done by comparing different grid sizes with various elements as presented in Table 2. The values of the Average Wall Shear Stress (WSS) on the sac surface with normal blood hematocrit value of HCT = 0.4 for four grids are compared in Table 2. A grid with 319,446 cells is selected as the final grid in this study. The main file of aneurysm shape is obtained from AneuriskWeb^46^.

**Table 2:** Grid study.

Element-size (mm)	Number of elements	Average of WSS on Sac (Pa)	Change %
0.28	168,074	12.73	–
0.23	227,168	14.97	17.59
0.18	319,446	15.84	5.81
0.15	678,147	15.88	0.25

## Results and discussion

**Figure 4:**
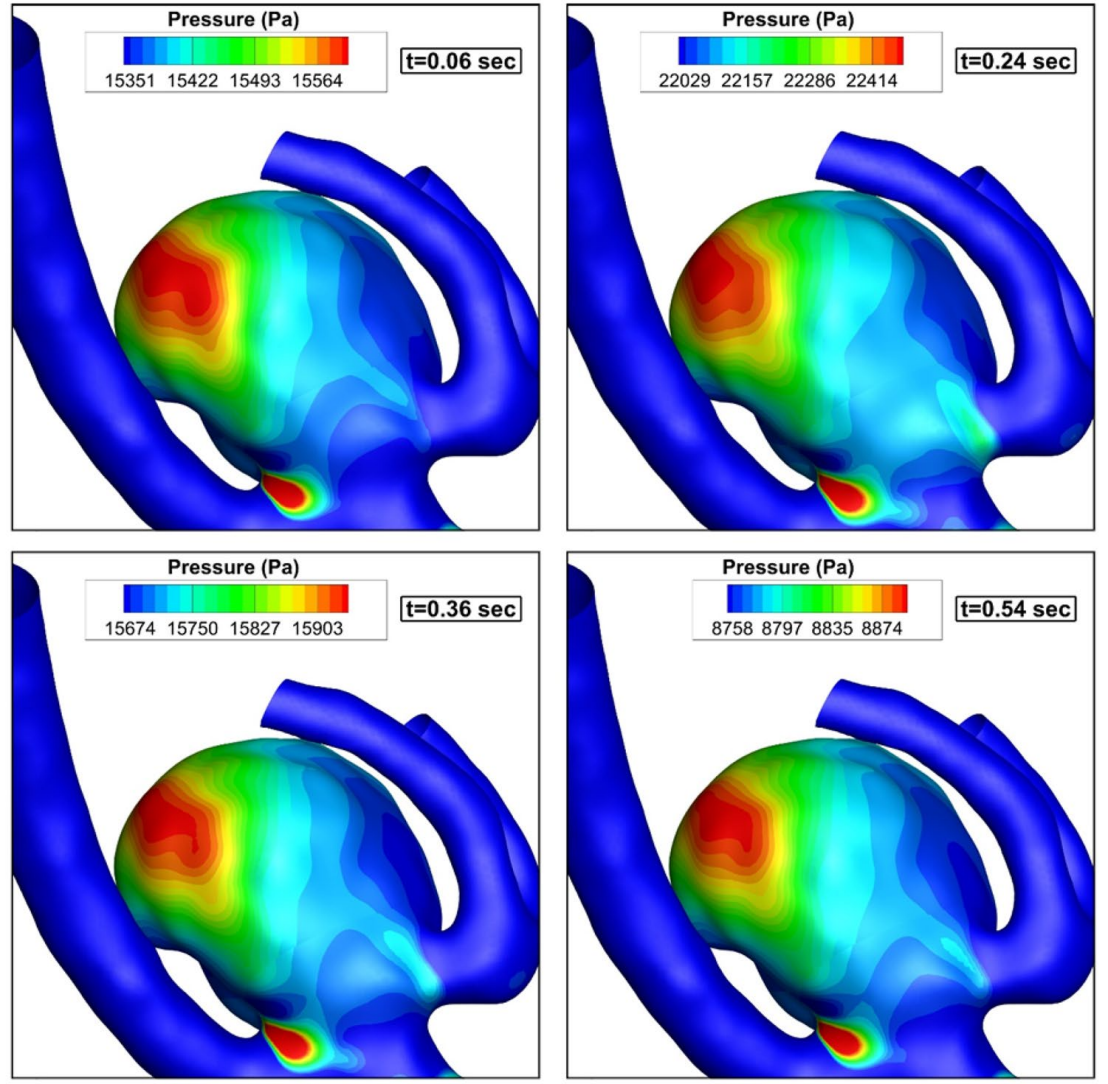
Pressure contour (hct = 0.40 without coiling (porosity = 1)).

**Figure 5:**
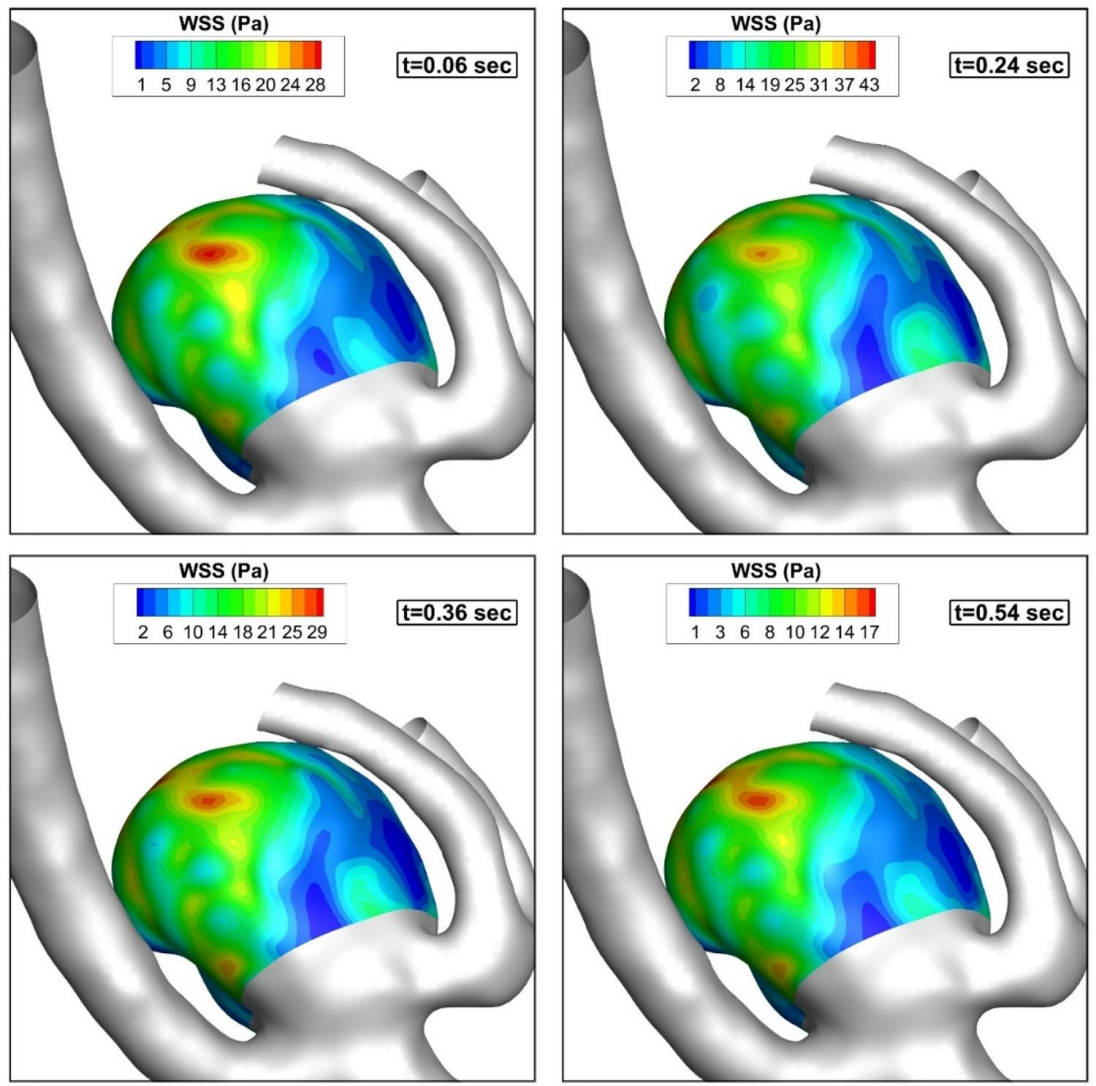
WSS contour (hct = 0.40 without coiling (porosity = 1)).

The pressure contour on the sac and vessel surface of the chosen MCA aneurysm at different stages of the cardiac cycle is presented in Fig. 4. The pressure contour indicated that the maximum pressure value is attained at t = 0.24 s when the acceleration of the incoming blood flow is extremely high. Figure 5 demonstrates the distribution of the WSS on the sac surface on the parent vessel and MCA sac. Due to the angle of incoming blood with the sac ostium, the maximum WSS is noticed near the sac dome due to the deflection of the blood stream.

**Figure 6:**
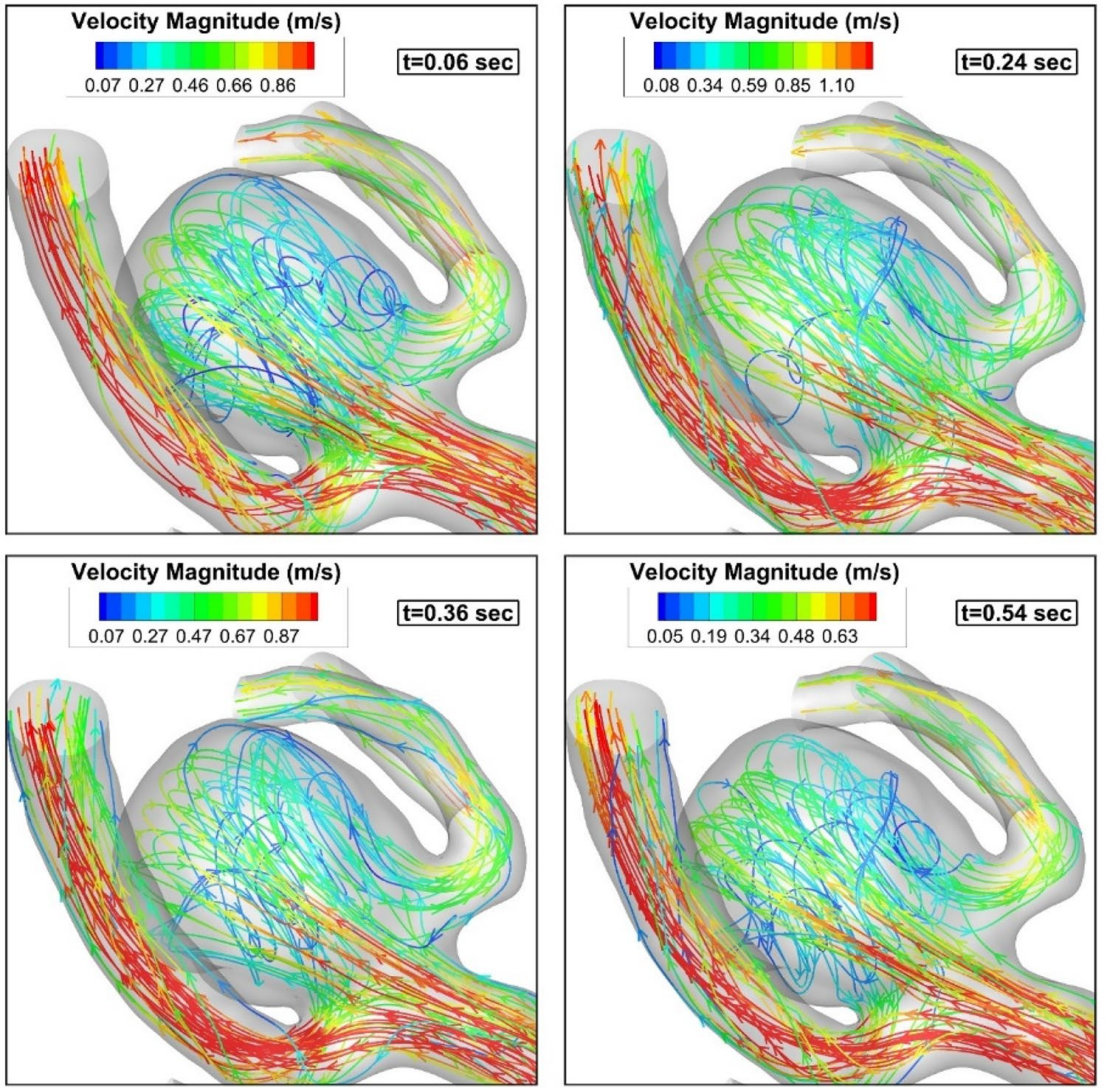
Blood stream.

**Figure 7:**
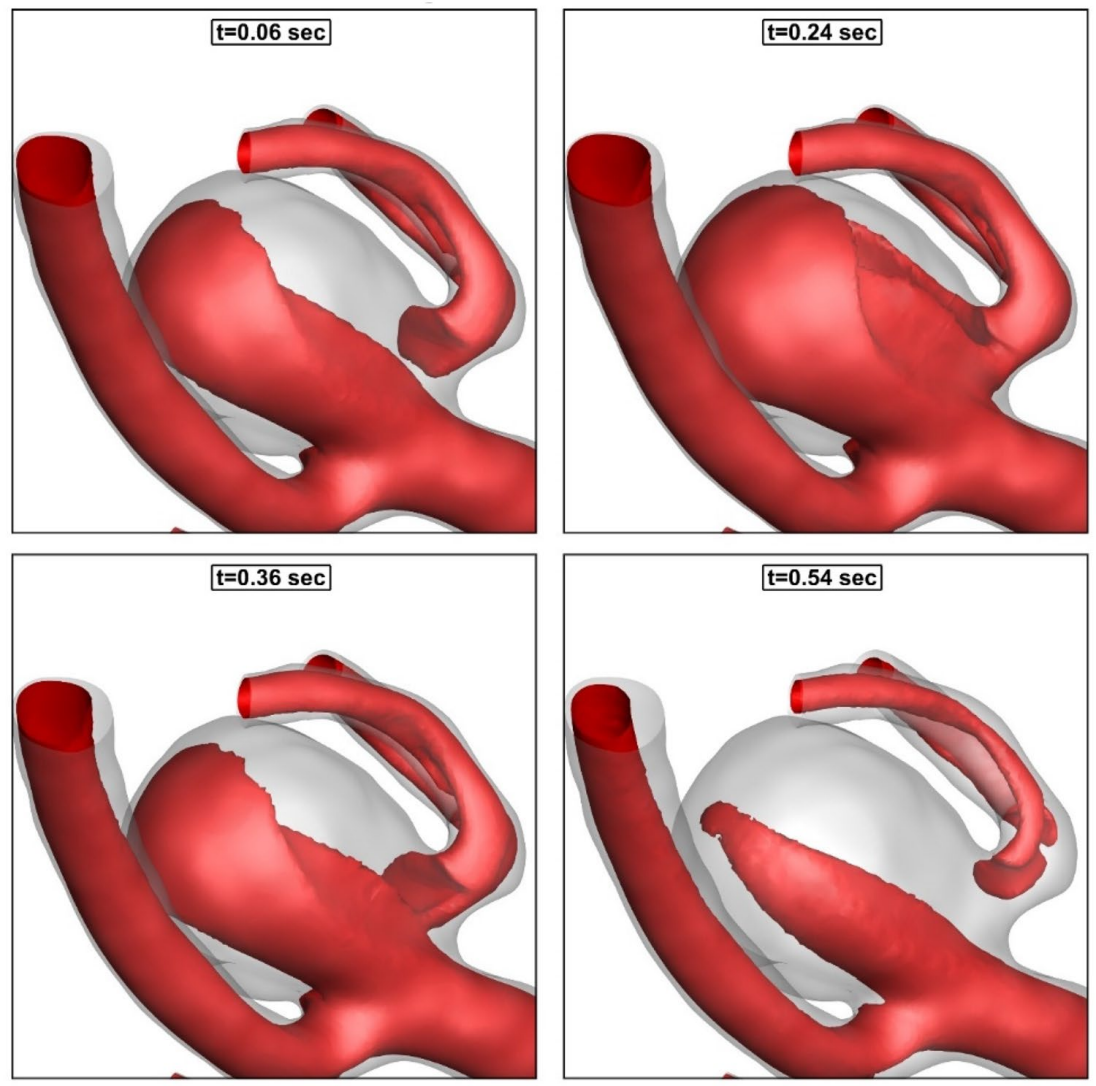
Iso-velocity (V = 0.5 m/s).

As demonstrated in Fig. 6, the incoming blood stream into the sac domain is deflected on the dome of an aneurysm while its velocity magnitude is reduced substantially in the sac domain. A comparison of the velocity magnitude in the blood stream confirms that blood flow has less velocity when it is deflected by the aneurysm dome. Figure 7 demonstrated the iso-velocity layer (v = 0.5 m/s) of blood to compare the blood hemodynamics at different stages of the cardiac cycle.

**Figure 8:**
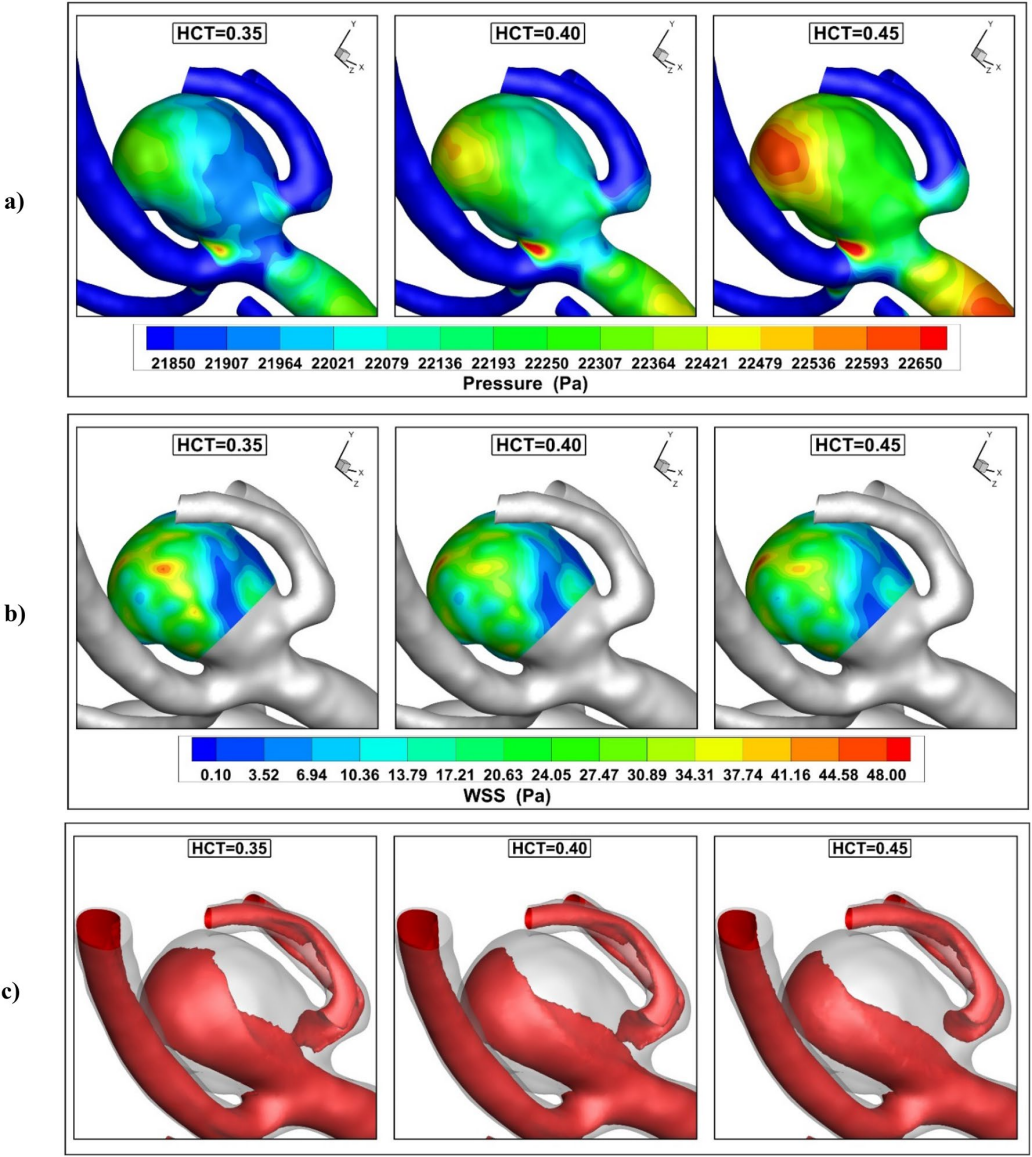
Influence of HCT on (**a**) pressure (**b**) AWSS (**c**) iso-velocity (v = 0.75 m/s) contour at peak systolic.

The influence of the blood hematocrit (HCT) on the pressure distribution on the sac surface is shown in Fig. 8a. Increasing the blood HCT substantially increases the HCT values on the sac surface at peak systolic (t = 0.24 s). A comparison of the WSS on the sac surface shows that the blood hematocrit is not effective on this factor (Fig. 8b). In addition, the hemodynamic feature of blood confirms that this factor does not change the flow hemodynamic.

**Figure 9:**
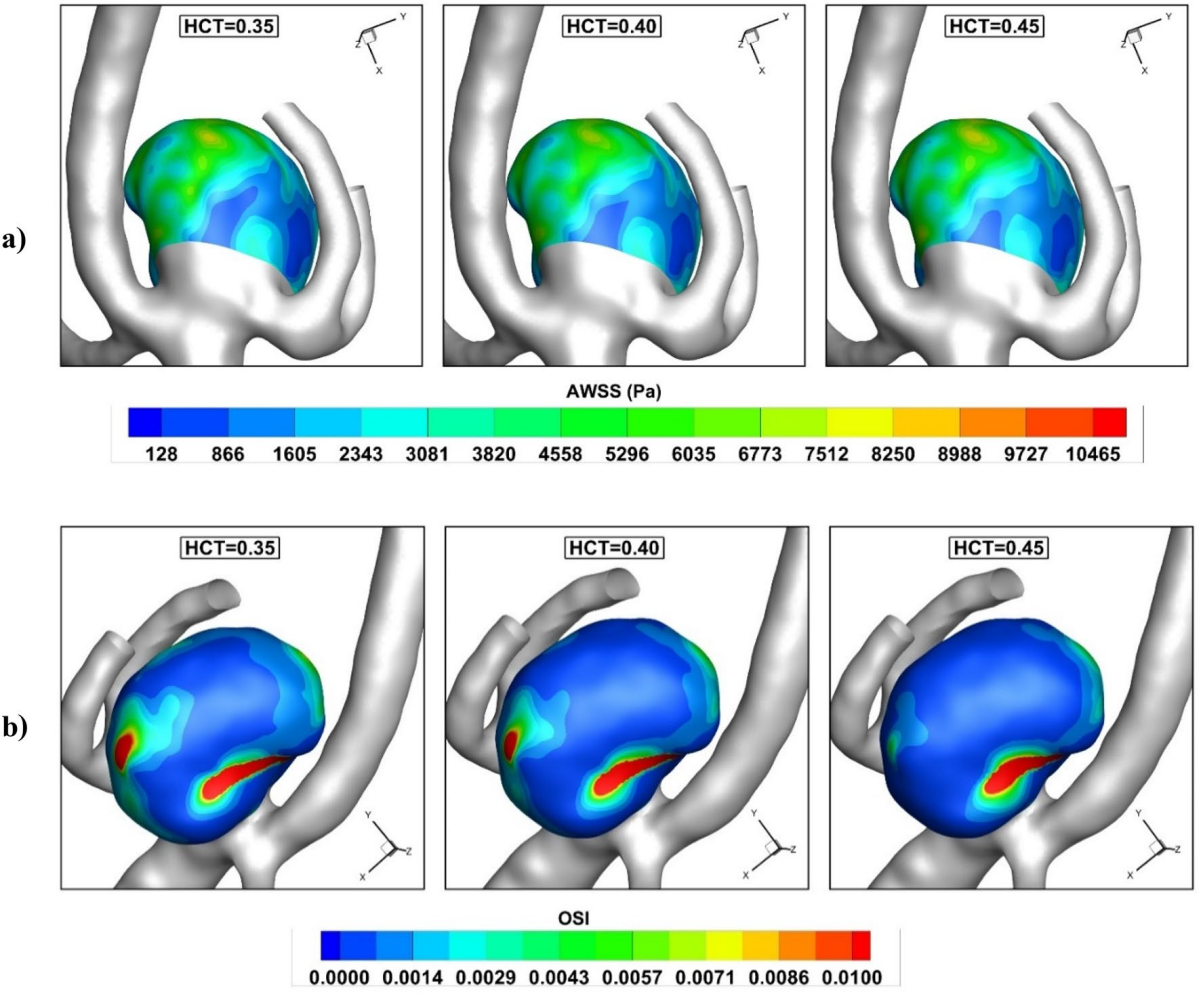
Influence of HCT on (**a**) pressure (**b**) AWSS (**c**) iso-velocity (v = 0.75 m/s) contour at early diastolic.

The variation of the AWSS and OSI index at early diastolic (t = 0.54 s) are displayed in Fig. 9. The critical region for probable rupture is located near ostium and neck region as demonstrated. In fact, due to the entrance and return of the blood flow at this section as well as the low area, the velocity of the blood is more than in other regions and this region is potential for the rupture.

**Figure 10:**
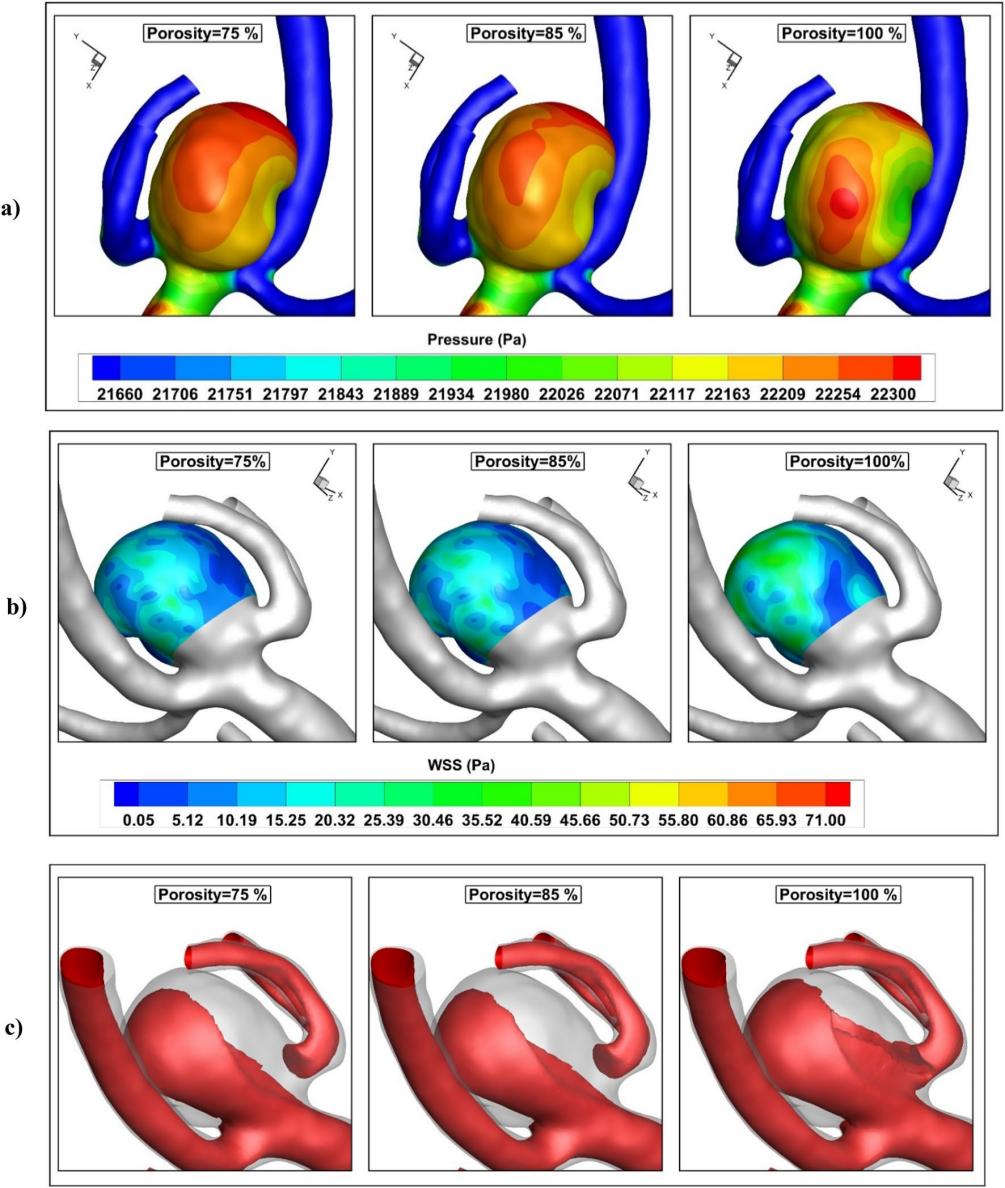
Influence of coiling porosity on (**a**) pressure (**b**) AWSS (**c**) iso-velocity (v = 0.6 m/s) contour at peak systolic.

The influence of coiling porosity on the distribution of the pressure, WSS, and blood hemodynamics at peak systolic are demonstrated in Fig. 10. The pressure distribution shows that increasing the coiling porosity decreases the pressure value on the sac dome. WSS distribution also confirms that the porosity of coiling increases the WSS on sac surface. The hemodynamic feature of the blood at different porosities (Fig. 10c) indicates that increasing the coiling porosity rises blood velocity near the wall.

**Figure 11:**
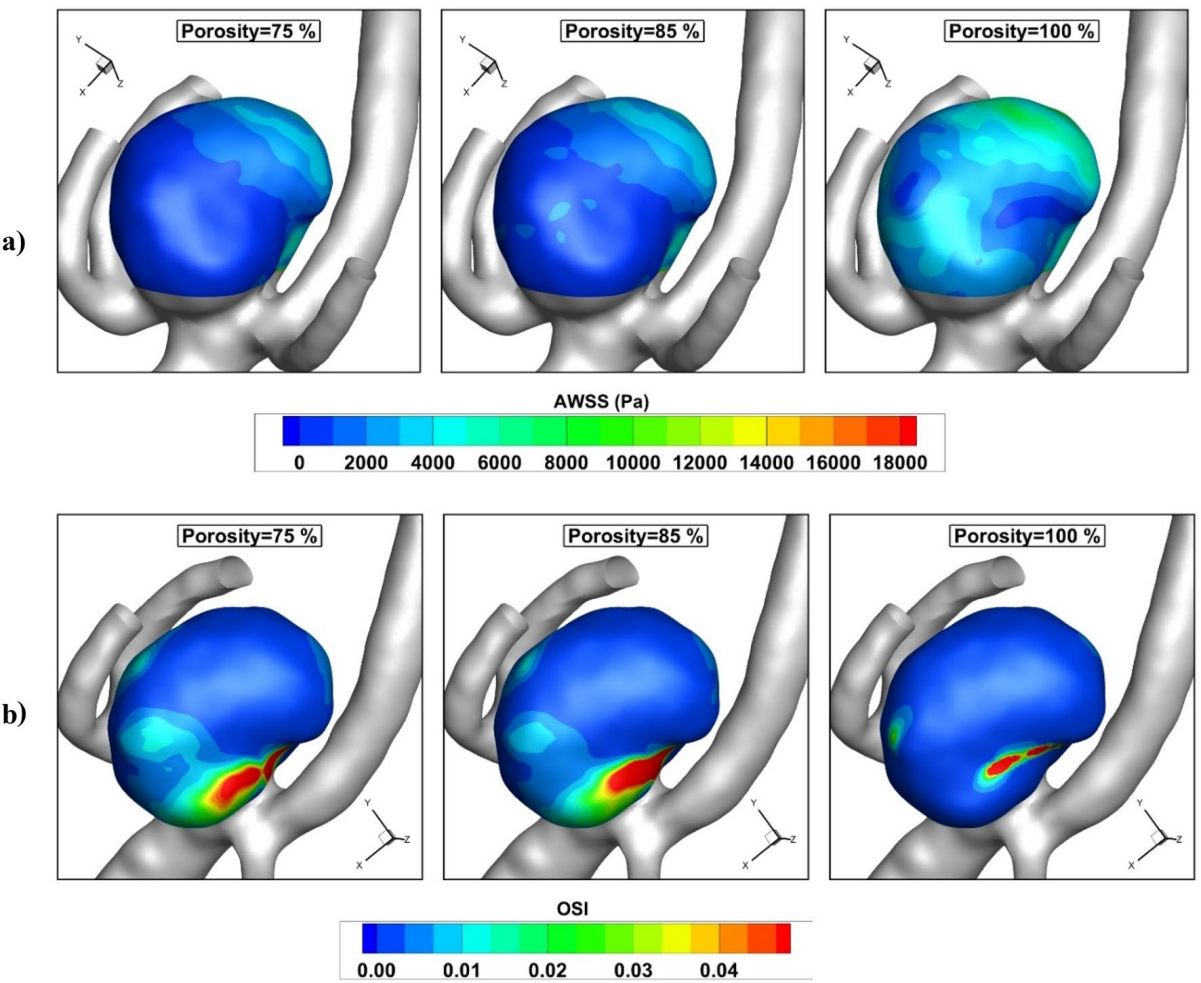
Influence of coiling porosity on (**a**) pressure (**b**) AWSS c) iso-velocity (v = 0.75 m/s) contour at peak systolic.

**Figure 12:**
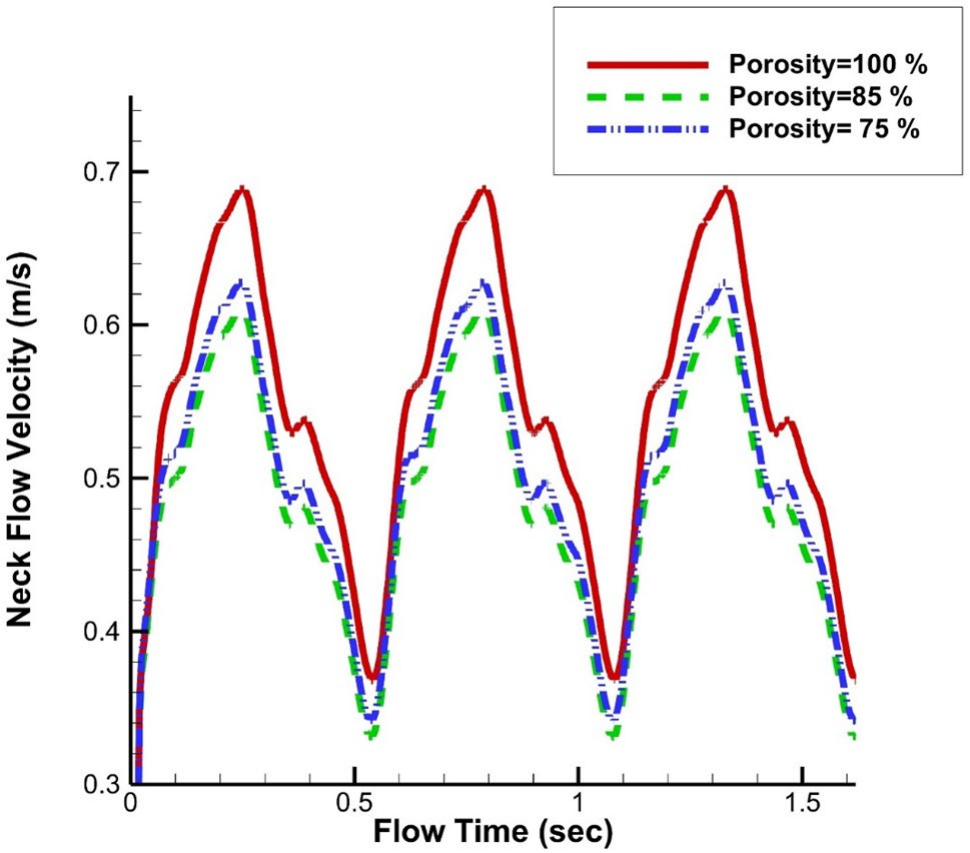
Variation of neck flow velocity in different coiling porosity.

Figure 11 demonstrates the impacts of coiling porosity on the AWSS and OSI at early diastolic (t = 0.54 s). The variation of the wall shear stress shows that the high-risk region remains near the aneurysm neck and the effects of coiling are limited in this section. The contour of OSI at the end of the cardiac cycle also represents the location of a high OSI value where the risk of rupture is high. Figure 12 demonstrates the variation of blood flow at the neck of an MCA aneurysm. The results of the fully porous domain (porosity = 100%) show that the velocity near the neck is high while we expected a lower value in this region. Although the maximum velocity of the blood at peak systolic is high for a fully porous domain, the minimum value of this case is higher than other cases. In fact, these models have identical velocity ranges.

**Figure 13:**
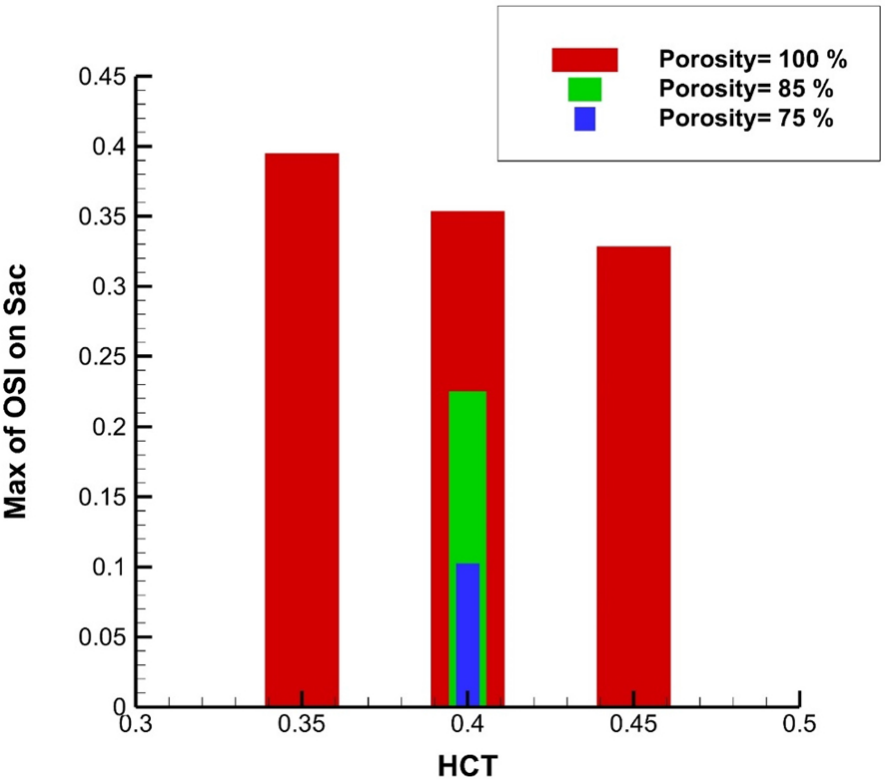
Maximum OSI on sac under impacts of coiling porosity.

**Figure 14:**
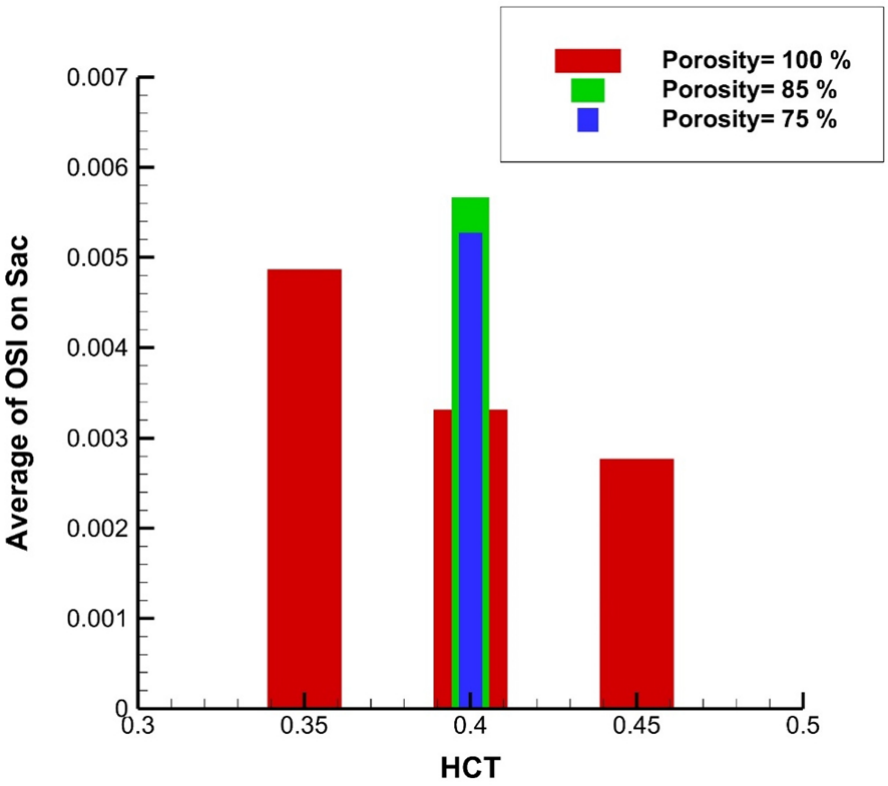
Average of OSI on sac under impacts of coiling porosity.

**Figure 15:**
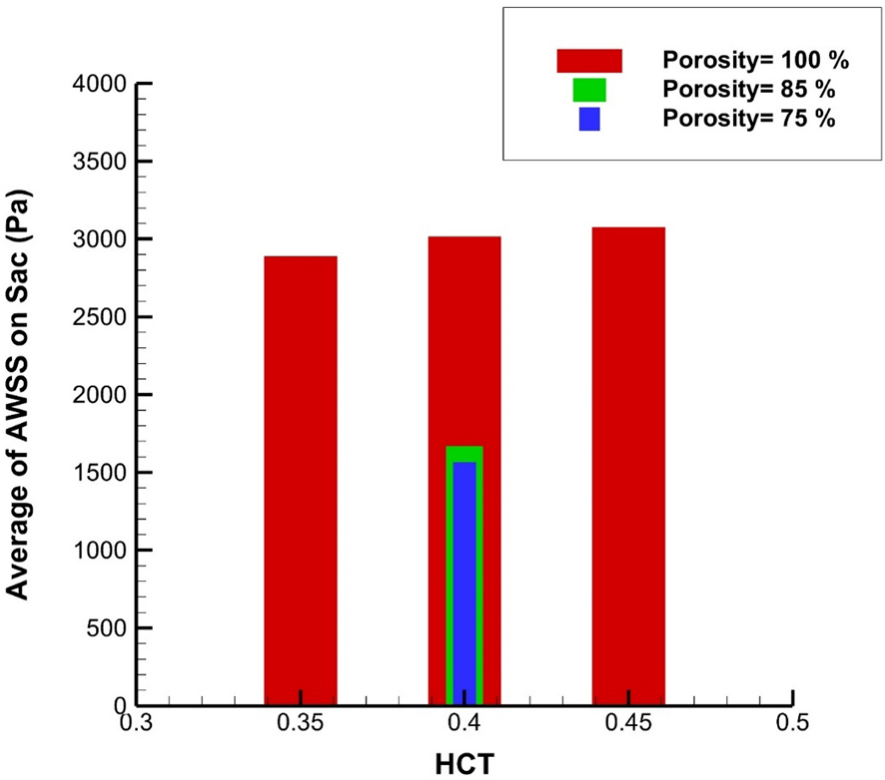
Average of WSS on sac under impacts of coiling porosity.

Figure 13 illustrates the Maximum OSI value at the sac section for different porosity and HCT ranges. Increasing the blood hematocrit (HCT) slightly decreases the Average OSI at end of the cardiac cycle. Decreasing the porosity values meaningfully increases Max OSI on sac wall. The results of average OSI at end of the cardiac cycle are presented in Fig. 14. Although the effects of HCT remain identical on the Maximum value of OSI (Fig. 14), the value of average OSI varies meaningfully by changing HCT value. Reducing the porosity significantly increases the average OSI value on the sac surface. Figure 15 demonstrates the impacts of HCT and coiling on the maximum AWSS. It is observed that the HCT effects are limited on the wall shear stress. However, the impact of the porosity is considerable on the shear rate of the aneurysm wall.

## Conclusion

In this study, computational fluid dynamics is used to investigate the blood hemodynamics on the risk-of MCA aneurysm rupture. Real saccular MCA aneurysm of the female patient is chosen in the present work. Navier–Stocks equations are solved for the simulation of blood stream moving inside the MCA aneurysm. The hemodynamic factors of pressure, WSS, and OSI are investigated in different blood hematocrit values. The impacts of the endovascular coiling are investigated by applying porosity inside the sac of the aneurysm. Hemodynamic factors are investigated at peak systolic and early diastolic as two main stages in the cardiac cycle. Achieved results show that reducing the porosity significantly increases the average OSI value on the sac surface. Besides, the HCT effects are limited on the wall shear stress.

## Data Availability

All data generated or analysed during this study are included in this published article.
